# Droplet to soliton crossover at negative temperature in presence of bi-periodic optical lattices

**DOI:** 10.1038/s41598-022-23026-x

**Published:** 2022-10-29

**Authors:** Maitri R. Pathak, Ajay Nath

**Affiliations:** Indian Institute of Information Technology Vadodara Gujarat India, Gandhinagar, 382 028 India

**Keywords:** Ultracold gases, Optics and photonics

## Abstract

It is shown that the phenomenon of negative temperature essentially occurs in Bose-Einstein condensate due to the realization of the upper bound energy state utilizing a combination of expulsive harmonic oscillator and optical lattice potentials. We study the existence of quantum droplets at negative temperature and droplet-to-soliton crossover in the binary Bose-Einstein condensate mixture in the presence of bi-periodic optical lattices and expulsive-BOL confinements. Based on the beyond mean field approximation, we employ the extended Gross-Pitäevskii equation and calculate the exact analytical form of wavefunction solutions for BOL, expulsive-BOL confinements. An interesting transition of quantum droplets from positive to negative temperatures and the droplet-to-soliton crossover by modulating the disorder in BOL potential are illustrated. The affirmation of such crossover is performed by exploring the profile of atomic condensate density which smoothly transits from being a flat top density in optical lattice confinement to a bright soliton for BOL trap. Further, we confirm the crossover by exploring the energy per particle and the variation in the root mean square size of the condensate with respect to the potential depth of the BOL trap. Eventually, all of this aid us to construct a phase diagram in a space between the amplitude of BOL potential depth and particle number which reveals the formation of droplet and soliton phases. In expulsive-BOL confinement, it is seen that the impact of the expulsive trap is insignificant on atomic condensate density in the droplet phase and it becomes prominent in the soliton region. Further, the variation of total energy reveals that the amplitude of the expulsive oscillator strengthens the droplet phase and leads to an increase in the negative temperature of the considered system.

## Introduction

A quantum droplet (QD) describes a self-bound many-body state which is stabilized in a regime where effective mean field interaction (EMF) can be made small and comparable to the beyond mean field (BMF) interaction generated due to the quantum fluctuation (Lee-Huang-Yang correction)^1,2^. In recent times, the theoretical and experimental investigations of QDs had received a lot of attention in the current ultracold literature, given its successful observation in dipolar gases^3–5^ and alkali binary Bose-Bose mixtures^6–8^. In a pioneering result, Petrov proposed that the weakly interacting binary Bose-Einstein condensate (BEC) mixture can be stabilized into a self-bound QD in free space by making a subtle balance between the repulsive cubic EMF and attractive quadratic BMF interactions in the one-dimensional (1D) geometry^9,10^. Before this, A. Bulgac suggested a mechanism for the existence of boselets, a droplet state based on the balance between two- and three-body interactions and the similar existence of fermilets, ferbolets are also theoretically predicted^11,12^. This balance of attraction and repulsion in ultracold atomic systems by taking into consideration the BMF quantum effects leads to the formation of self-bound ultra-dilute quantum liquid droplets of fermionic or bosonic atoms even in the absence of high atomic density and van der Waal’s interactions. These ultra dilute QDs provide an ideal platform for the investigation of beyond mean field quantum exotic phases and the research in this field is bringing out various interesting physical phenomena: droplet to soliton transition^13^, supersolid behavior in dipolar droplets^14,15^, formation of striped states in QDs^16^, vortices generation^17^, the possibility of spinor boson droplets^18^ and, fragmentation of droplets^19^ and its clusters^20^.

In this work, we study the generation and dynamics of QDs at a negative temperature in the presence of a bi-periodic optical lattice (BOL) and an expulsive-BOL superlattice. Negative absolute temperatures were first investigated in point vortices by Onsager^21^ and the field has been intensively explored since 1951 when Purcell first observed it in nuclear spin systems^22^. In 1956, Ramsey proposed these states as thermodynamic equilibrium states^23^. Based on the thermodynamics law, the existence of negative temperature (*T*) $[T = (\partial S/\partial E)^{-1}]$ state is feasible if entropy (*S*) does not increase monotonically with energy (*E*) and reaches its maximum away from the domain boundary^24^. The essential requirement for reaching negative temperature in a physical system is that the energy levels of the system need to be an upper bound. In the field of BEC, Braun *et. al.* using weakly interacting attractive $^{39}K$ atoms realized negative temperature motional states by creating upper energy bound for the combination of anti-trap (inverted parabolic oscillator) and optical lattice confinements^24,25^. Out of these potentials, inverted parabolic confinement provides the upper bound in potential energy, bands of periodic trap ensure the upper bound in kinetic energy, and the attractive nonlinearity between atoms makes the interaction energy upper bound. The experimental observation of negative temperature received a lot of criticism and some works associate negative temperature with non-equilibrium states based on the definition of entropy^26,27^. Despite this, negative temperatures are now well accepted by a large community and the results of experiments appear to be well-grounded^28–31^. Recently, an exact analytical model for the dynamics of solitons in cigar-shaped BEC at negative temperature using a combination of expulsive harmonic oscillator-BOL superlattice confinements is constructed which showed that the oscillator frequency and potential depth of BOL trap provide tunability on the negative temperature of the system^32^. Kundu et al. illustrated that the BOL trap alone (without combining with an expulsive trap) is capable of creating the negative temperature domain for a quasi-1D BEC where frustration depth of BOL acts as upper bound energy level^33^. Further, negative temperature has been investigated for autonomous refrigeration of qubits^34^, work storage^35^, cosmology^36^, quantum fluctuations^31^, Carnot engine^37^ etc. Although, in the last few years, droplets are extensively investigated in free space region i.e. without any external confinement^1,2,38–40^ and an exact theoretical model for QDs in harmonic and multi-color optical lattice confinements is also recently reported^41,42^. However, the generation and dynamics of QDs are not investigated in the negative temperature regime. The main reason lies in the fact that exploring the exact droplet dynamics under such a scenario is analytically quite nontrivial. Moreover, quantifying the negative absolute temperature and correlating it with viable trap parameters will reveal new droplet domain physics.

In the following section, we present an analytical framework for investigating the droplets dynamics by constructing an exact solution of 1D extended Gross-Pitäevskii equation (eGPE) at a negative temperature under the BOL and expulsive harmonic oscillator-BOL confinements in the presence of competing repulsive cubic EMF and attractive quadratic BMF interactions. Based on the beyond mean field consideration, the droplet dynamics are modeled by utilizing the 1D eGPE in the presence of the above confinements which are suitable for the experimental realization of negative temperature. Here, we have considered the cigar-shaped two-component BEC mixture with an equal number of atoms in each component and having equal inter-atomic coupling constants describing the repulsion between the atoms. The motivation for choosing the combination of expulsive harmonic oscillator and BOL superlattice for studying droplet dynamics at negative temperature is twofold: (i) BOL as a quantum simulator: BOL confinements are quasi-periodic which are extensively utilized to study Anderson localization and a variety of quasi- periodic physical phenomena in BEC^43–45^. Further, the negative temperature state is shown to exist in BOL and expulsive-BOL confinements^32,33^; (ii) study novel phase transition: recently, Morera et al. investigated droplet phase in optical lattice confinement by tuning the inter-atomic interactions and number of particles in the binary bose-bose BEC mixture^46,47^. Additionally, the supersolid-like crystallization of QDs in a pancake-trapped dipolar BEC is explored in the optical lattice confinement^48,49^. Motivated by that, here, we calculate the exact analytical form of the wavefunction, EMF/BMF nonlinearities, and phase for 1D eGPE in the presence of considered expulsive-BOL traps. It is shown that with suitable choice of the expulsive oscillator frequency, one can realize the following experimentally realizable forms of trap configurations: (a) free space; (No confinement); (b) $V(x,t)=V_{1} cos(2kx) + V_{2} cos(kx)$ (space-dependent BOL); and (c) $V(x,t)=V_{1}(t) cos(2kx) + V_{2}(t) cos(kx)-\frac{1}{2}M^2 x^2$; (space- and time-dependent expulsive-BOL) and observe negative temperature scenario. Here, $V_{1}$, $V_{2}$, $V_{1}(t)$, $V_{2}(t)$ are potential depths of interfering optical lattices, *M* and *k* are expulsive oscillator frequency and optical lattice vector, respectively. Interestingly, the consistency conditions governing the expulsive oscillator frequency maps with the linear Schrödinger equation which presents an important coherent control over the droplet dynamics. Next, we investigate the dynamics of droplets under the influence of BOL and expulsive-BOL traps. Utilizing the atomic density analysis, we illustrate an interesting transition of positive to negative temperature and droplet-soliton crossover by modulating the disorder and symmetry of the BOL trap. The affirmation of such crossover is performed by exploring the Anderson-like localization of atomic condensate density profile which smoothly transits from being a flat top density in free space to a bright soliton for BOL confinement. For supplementing the droplet to soliton crossover, we explore the energy per particle and the root mean square size of the condensate bound pairs for the changing potential depth and symmetry of the BOL trap and confirm the crossover. Using this, we construct a phase diagram in a space between the amplitude of BOL potential depth and particle number which reveals the formation of droplet and soliton phases. Lastly, we observe the impact of the expulsive-BOL trap combination on the droplet-soliton crossover and the transition of positive to negative temperature in comparison to only BOL confinement by tuning the expulsive oscillator strength. From the atomic condensate density profile, it is seen that expulsive trap impact is insignificant in the droplet phase, however, it becomes prominent in the soliton region. It is observed that the increase in the magnitude of expulsive oscillator strength results in the increase of bound energy in the droplet phase and simultaneously leads to an increase in the negative temperature of the system.

## Results

### Exact analytical model for negative temperature

We consider the 1D mass-balanced binary BEC mixture with mutually symmetric spinor components in the presence of an expulsive harmonic oscillator and BOL confinements. Further, we assume that the inter-atomic coupling constants controlling the repulsion between the atoms in each component are identical (i.e. $g_{11} = g_{22}$) such that the equilibrium densities of both components are equal. In the 1D geometry, the droplet dynamics are described by the underlying time-dependent eGPE^10^:

1 $$\begin{aligned} i \hbar \frac{\partial \psi _1}{\partial t} + \frac{\hbar ^2}{2m}\frac{\partial ^2\psi _1}{\partial z^2} - (\chi _s(z, t) |\psi _1|^2 + \chi _c(z, t) |\psi _2|^2)\psi _1 + \Lambda (z, t) (|\psi _1|^2+|\psi _2|^2)^{1/2}\psi _1-v(z, t) \psi _1=0, \end{aligned}$$

2 $$\begin{aligned} i \hbar \frac{\partial \psi _2}{\partial t} + \frac{\hbar ^2}{2m}\frac{\partial ^2\psi _2}{\partial z^2} - (\chi _c (z, t) |\psi _1|^2 + \chi _s (z, t) |\psi _2|^2)\psi _2 + \Lambda (z, t)(|\psi _1|^2+|\psi _2|^2)^{1/2}\psi _2 -v(z, t) \psi _2 =0, \end{aligned}$$

where $\psi _1$ ($\psi _2$) represents the first (second) component wavefunctions of the binary BEC mixture and *v*(*z*, *t*) is the chosen external confinement. Here, $g_{11} = g_{22}\equiv g=2 \hbar ^{2} a_{s}(z, t)/(m a^{2}_{\perp })$ and $g_{c}= g_{12}$ with $a_{\perp }$ and $a_{s}(z, t)$ are the transverse oscillator length and the inter- and intra- components atomic scattering lengths, respectively. In the above equations, $\chi _s (z, t)=(g_{c}+3g)/2$ represents the self interaction coefficients whereas $\chi _c (z, t)=(g_{c}-g)/2$ is the cross interaction coefficients along with $\Lambda (z, t)=\sqrt{m}g^{3/2}/(\pi \hbar )$^41,42^. The sign and strength of both: $\chi _c (z, t)$ and $\Lambda (z, t)$ are dependent on the inter- and intra-components atomic scattering lengths. Experimentally, these lengths are tunable using the Feshbach resonance technique^50^ which provides control over the intra- and inter-component inter-atomic interactions. Here, $\hbar$ is scaled by Planck’s constant and *m* is the mass of the BEC atom.

Considering $\psi _{1}=\psi _{2}=\psi _{0} \psi$ and substituting it in the above Eqs. (1) and (2). This reduces it to the following 1D dimensionless single eGPE^9,10^:

3 $$\begin{aligned} i\frac{ \partial \psi }{\partial t} = -\frac{1}{2} \frac{ \partial ^2 \psi }{\partial z^2} - g_1(z, t) |\psi | \psi + g_2(z, t)|\psi |^2 \psi + V(z, t) \psi , \end{aligned}$$

with $\psi (z, t)$ denote the condensate wave function. $g_{1}(z, t)=\Lambda (z, t)$ and $g_{2}(z, t)=\chi _{s}(z, t) + \chi _{c}(x, t)$ are the inter-atomic interaction strengths of two-component BEC mixture representing BMF and EMF interactions, respectively. Equation (3) is the extended form of Gross-Piteäevskii equation added with external confinement which is utilized to investigate droplet dynamics for various physical scenarios^40–42^. Here, *V*(*z*, *t*) is the external confinement, which is taken as the combination of an expulsive harmonic oscillator, BOL and exponential periodic confinements for obtaining negative temperature scenario^25,32,33^:

4 $$\begin{aligned} V(z,t)=-\frac{1}{2}M(t)z^{2}+V_{1}(t)\cos (2 k \, \xi ) + V_{2}(t) \cos (k \, \xi ) + \frac{1}{2} E \gamma ^{2}(t) exp[2 \, \beta \, cos(k \, \xi )]. \end{aligned}$$

Here, $\xi (z, t)= \gamma (t) z$, with $\gamma (t) \ne 0$. *M*(*t*) is the time-dependent harmonic oscillator frequency, and $V_{1}(t)$, $V_{2}(t)$ are the potential depths of the BOL trap which is usually expressed in terms of the recoil energy^45^, respectively. Experimentally, BOL potential depths can be controlled through the wavelength of the laser ($\lambda$) and the mass of the BEC atoms. In Eq. (4), the frequency of the two laser beams are commensurate and $k = 2 \pi a_{\perp }/ \lambda$ is the scaled lattice wave vector. For $-1<\beta <1$, it becomes:

5 $$\begin{aligned} V(z,t) \simeq - \frac{1}{2}M(t)z^{2}+ V_{1}(t) \cos (2 k \, \xi ) + \left[ V_{2}(t) + E \beta \gamma ^{2}(t)\right] \cos (k \, \xi ). \end{aligned}$$

From Eqs. (4) and (5), depending on the magnitude of $\beta$, the trap profile is: (a) expulsive harmonic oscillator and BOL for $-1< \beta < 1$; and (b) for $\beta$ $>1$ or $<-1$, the trap profile is the combination of expulsive harmonic oscillator and multi-color optical lattice which contains complete series of Fourier harmonics^42^. Now, to construct the analytical solution of $\psi$ for Eq. (3), following the general similarity transformation scheme used for solving GPE^51–53^, we start with an ansatz solution:

6 $$\begin{aligned} \psi (z, t)=A(z, t) U[\xi (z, t)]exp[i\phi (x,t)], \end{aligned}$$

where *A*(*z*, *t*), $\phi (z,t)$ and $\xi (z,t)$ are the amplitude, phase and travelling coordinate (similarity variable) which are space- and time-dependent, respectively. Using the ansatz solution, our objective is to connect Eq. (3) with the following differential equation

7 $$\begin{aligned} -\frac{\partial ^2 U}{\partial \xi ^2}-G_1\mid U(\xi )\mid U +G_2\mid U(\xi )\mid ^{2} U=E U, \end{aligned}$$

where *E* is the eigenvalue of equation and $G_1$, $G_2$ are the BMF, EMF interactions constant, respectively. $G_1$, $G_2$ can take positive or negative magnitude depending on the sign of inter- and intra- component atomic scattering lengths. This results in the following set of constraints on the amplitude, phase, EMF, and BMF nonlinearities:

8 $$\begin{aligned}{}[A^{2}(z,t)\xi _{z}(z,t)]_{z}=0, \,\,\,\,\,\, \xi _{t}(z,t)+\xi _{z}(z,t)\phi _{z}(z,t)=0, \end{aligned}$$

9 $$\begin{aligned} G_{1} \xi _{z}^{2}(z,t)-2 A(z,t) g_{1}(z,t)=0, \,\, G_{2} \xi _{z}^{2}(z,t)-2 A^{2}(z,t) g_{2}(z,t)=0, \end{aligned}$$

10 $$\begin{aligned} \frac{A_{t}(z,t)}{A(z,t)}+\frac{1}{2 A^{2}(z,t}[A^{2}(z,t)\phi _{z}(z,t)]_{z}=0, \end{aligned}$$

11 $$\begin{aligned} \frac{A_{zz}(z,t)}{2A(z,t)}-\frac{\phi _{z}^{2}(z,t)}{2}-\phi _{t}(z,t)-\frac{1}{2} E \xi _{z}^{2}(z,t)-V(z,t)=0. \end{aligned}$$

In the above equation, the function with subscript infers the partial differentiation of the function with respect to the subscripted variable. The above set of consistency conditions are simultaneously solved to obtain amplitude, phase, EMF, and BMF nonlinearities:

12 $$\begin{aligned} A(z,t)= \sqrt{\frac{c(t)}{\xi _{z}(z,t)}}, \,\, \phi _{z}(z,t)=-\frac{\xi _{t}(z,t)}{\xi _{z}(z,t)}, \,\, g_{1}(z,t) = G_{1} \frac{\xi _{z}^{2}(z,t)}{2 A(z,t)}, \,\, g_{2}(z,t) = G_{2} \frac{\xi _{z}^{2}(z,t)}{2 A^{2}(z,t)}, \end{aligned}$$

where *c*(*t*) is an integration constant. It is clear from the Eq. (12) that the form of phase and EMF, BMF nonlinearities are proportional to the amplitude (*A*(*z*, *t*)). The form of *A*(*z*, *t*) is dependent on the choice of external confinement and it will be calculated by solving the consistency equation (11). For that purpose, we substitute the trap expression from Eq. (4) into the set of consistency Eqs. (8) and (12) and choose $\xi (x,t)= \int _{0}^{\xi } exp[\beta \cos (k \xi )] \partial \xi '$, to obtain the exact analytical form of amplitude, phase and nonlinearities:

13 $$\begin{aligned} A(z,t)=\sqrt{\frac{c(t)}{\gamma (t) \times exp[\beta \cos (k \xi )]}},\,\, \theta (x,t)=- \frac{\gamma '(t)}{2\gamma (t)} z^{2} + \left[ \frac{\beta ^{2} k^{2} \gamma ^{2}(t)}{16} \right] t, \end{aligned}$$

14 $$\begin{aligned} g_{1}(x,t)=\frac{G_{1} \gamma ^{3/2(t)}}{2 c(t)} exp[\beta \cos (k \xi )]^{\frac{3}{2}},\,\, g_{2}(x,t)=\frac{G_{2} \gamma ^{3}(t)}{2 c(t)}exp[\beta \cos (k \xi )]^{3}, \end{aligned}$$

with the potential depths of two optical lattices forming BOL are connected in the following manner: $M(t)=-(8 \gamma (t) \gamma _{tt}(t)-16 \gamma _{t}^{2}(t)) /(16 \gamma ^{2}(t))$, $V_{1}(t) =-[\beta ^{2} k^{2} \gamma ^{2}(t)]/16$,    $V_{2}(t)=[\beta k^{2} \gamma ^{2}(t)]/4$. It results in making the final form of confinement:

15 $$\begin{aligned} V(z,t)= & {} - \frac{1}{2} \left[ \frac{8 \gamma (t) \gamma _{tt}(t)-16 \gamma _{t}^{2}(t)}{16 \gamma ^{2}(t)} \right] z^{2}+\left[ -\frac{\beta ^{2} k^{2} \gamma ^{2}(t)}{16} \right] \cos (2 k \, \xi ) \nonumber \\{} & {} + \left[ \frac{\beta k^{2} \gamma ^{2}(t)}{4} \right] \cos (k \, \xi ) + \frac{1}{2} E \gamma ^{2}(t) exp[2 \, \beta \, cos(k \, \xi )], \,\,\,\,\, (\beta < -1, \beta >1) \end{aligned}$$

16 $$\begin{aligned} V(z,t)\simeq & {} - \frac{1}{2} \left[ \frac{8 \gamma (t) \gamma _{tt}(t)-16 \gamma _{t}^{2}(t)}{16 \gamma ^{2}(t)} \right] z^{2}+ \left[ -\frac{\beta ^{2} k^{2} \gamma ^{2}(t)}{16} \right] \cos (2 k \, \xi ) \nonumber \\{} & {} + \left[ \frac{\beta k^{2} \gamma ^{2}(t)}{4} + E \beta \gamma ^{2}(t)\right] \cos (k \, \xi ) \,\,\,\,\, (-1< \beta < 1). \end{aligned}$$

Equation (15) reveals each coefficients of time-dependent expulsive-BOL confinement. It is worth to note here that the form of *M*(*t*) for $\gamma (t)=\frac{1}{\omega (t)}$ transforms into $\omega _{tt}(t)+2\omega (t)M(t)=0$, which is well-known linear Schrödinger equation. Further, the transformation, $\omega (t)=e^{-\int _{0}^{t}\nu (t')dt'}$, allows transformed Schrödinger equation to be written as the Riccati equation, $\nu _{t}(t)-\nu ^{2}(t)=2M(t)$. Taking advantage of these connections, corresponding to each solvable quantum-mechanical system, one can identify a droplet configuration. Thus, these equations provide an analytical control to tune the dynamics of the droplets in many ways as the solutions of Schrödinger equation and the Riccati equation can be obtained for various forms of *M*(*t*). However, in order to focus on the main goal of this paper for realizing the negative temperature scenario, we take the form of the $\gamma (t) = \gamma _{0} Sech(M t)$ with $\gamma _{0} \ne 0$ and $-1 <\beta<$ 1, such that the potential becomes:

17 $$\begin{aligned} V(z,t) \simeq -\frac{1}{2} M^{2} z^{2}+ \gamma ^{2}_{0} Sech^{2}(M t) \left[ -\frac{\beta ^{2} k^{2} }{16} \right] \cos (2 k \, \xi ) + \gamma ^{2}_{0} Sech^{2}(M t) \left[ \frac{\beta k^{2}}{4} + E \beta \right] \cos (k \, \xi ). \end{aligned}$$

The above potential can produce the following physical situations:

- For $M=0$, and $\beta =0$ (free space);
- For $M=0$, and $\beta \ne 0$ (space-dependent BOL); and
- For $M \ne 0$, and $\beta \ne 0$ (space- and time-dependent expulsive-BOL).

In principle, by modulating the magnitude of $\beta$ and *M*, one can tune the potential depths, period, and symmetry of the traps and obtain the above three forms of confinements out of which the last two are useful for the realization of negative temperature. Now, utilizing Eq. (13), we can write the complete solution of Eq. (3) as:

18 $$\begin{aligned} \psi (z,t)= & {} \sqrt{\frac{\gamma (t)}{exp[\beta \cos (k\,\, \xi )]}} \frac{\frac{3 E}{G_{1}} }{1+\sqrt{1-\frac{E}{\mu _{0}} \frac{ G_{2}}{ G_{1}^{2}} } \cosh (\sqrt{\text {-E}} (\int _{0}^{\xi } exp[\beta \cos (k \xi )] \partial \xi '))} \nonumber \\{} & {} \times exp \left[ i ( - \frac{\gamma '(t)}{2\gamma (t)} z^{2} + \left[ \frac{\beta ^{2} k^{2} \gamma ^{2}(t)}{16} \right] t) \right] , \end{aligned}$$

where $c(t)= \gamma ^{2}(t)$ with $\mu _{0}=-2/9$, $-2/9 \le E<0$, $G_1<0$ and $G_2>0$. Here, we considered the solution of Eq. (7) as: $U[\xi ]=\frac{3 (E/G_{1}) }{1+\sqrt{1-\frac{E}{\mu _{0}} \frac{ G_{2}}{ G_{1}^{2}} } \cosh (\sqrt{\text {-E}}\xi )}$^9,40^. Now, using Eq. (18), we calculate the normalization *N* in presence of expulsive-BOL confinement as:

19 $$\begin{aligned} N=\int _{-\infty }^{+\infty }|\psi |^2 \partial z=\frac{4}{3} \left[ ln \left( \frac{1+\sqrt{\frac{E}{\mu _{0}}}}{\sqrt{1-\frac{E}{\mu _{0}}}}\right) -\sqrt{\frac{E}{\mu _{0}}}\right] . \end{aligned}$$

The variation of *dN*/*dE* with respect to *E* in the domain $[-2/9, 0]$ turns out to be greater than zero which indicates the stable nature of the obtained solution as per the Vakhitov-Kolokolov criterion^54^. Thus, we constructed a large family of droplet solutions for 1D eGPE Eq. (3) for expulsive harmonic oscillator and BOL confinements in this section. In the upcoming sections, we will utilize it for investigating the droplet dynamics at negative temperature.

### Generation of quantum droplets in BOL at negative temperature

In this section, we investigate the generation of QDs and droplet-to-soliton transition in the presence of the space-dependent BOL trap. For that purpose, we take $M=0$, $\gamma _{0}=1$, and $\beta \ne 0$ in potential Eq. (17) which results into the BOL potential: $V(z,t) \simeq \left[ -( \beta ^{2} k^{2})/16 \right] \cos (2 k \, \xi ) + \left[ (\beta k^{2}/4) + E \beta \right] \cos (k \, \xi )$, with $\xi (z,t)=z$ and $-1<\beta <1$. The corresponding BMF and EMF nonlinearities takes the form: $g_{1}(x,t)= (G_{1}/2) exp[\beta \cos (k \xi )]^{\frac{3}{2}}$, $g_{2}(x,t)=(G_{2}/2 ) exp[\beta \cos (k \xi )]^{3}$, respectively. Thus, utilizing Eq. (18), the complete form of the wavefunction can be written as:

20 $$\begin{aligned} \psi (z,t)=\sqrt{\frac{1}{exp[\beta \cos (k\,\, z)]}} \frac{\frac{3 E}{G_{1}} }{1+\sqrt{1-\frac{E}{\mu _{0}} \frac{ G_{2}}{ G_{1}^{2}} } \cosh (\sqrt{\text {-E}} (\int _{0}^{z} exp[\beta \cos (k z)] \partial z'))} \times exp \left[ i \left( \frac{\beta ^{2} k^{2}}{16} \right) t \right] , \end{aligned}$$

where $$\mu _{0}=-2/9$$, $$E<0$$, $$G_1<0$$ and $$G_2>0$$.

**Figure 1:**
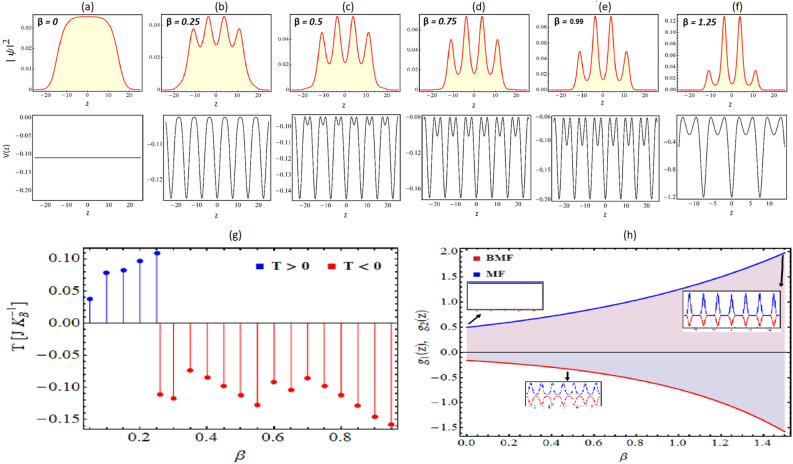
Condensate density patterns and corresponding BOL trap with $M = 0$ and $\beta \ne 0$: (**a**) $\beta = 0$ (free space); (**b**) $\beta = 0.25$, (**c**) $\beta = 0.5$, (**d**) $\beta = 0.75 0$, (**e**) $\beta = 0.99$, and (**f**) $\beta =1.25$. Each plot of (**a**–**f**) has two panels: the upper panel shows the density plot, and the lower panel indicates the corresponding trap profile. (**g**) The figure depicts the estimate of temperature ($J K_{B}^{-1}$) with respect to the potential depth of BOL ($\beta$) and illustrate positive to negative temperature transition. (**h**) The profile of EMF and BMF is plotted with changing $\beta$. Here, the magnitude of physical parameters: $\gamma _{0}=1$, $k=0.84$, $G_{1}=-1$, $G_{2}=0.999999$, $E=-2/9$. The spatial co-ordinate is scaled by the oscillator length. Figures are plotted using MATLAB R2020b (Master License 31349846) and Mathematica version 1.5.1.2021061827, Wolfram Research, Inc.

In Fig. 1(a–f), we illustrate the variation of condensate atomic density profiles with respect to the changing magnitude of BOL potential depth with $M = 0$ and $\beta > 0$: (a) $\beta = 0$ (free space); (b) $\beta = 0.25$, (c) $\beta = 0.5$, (d) $\beta = 0.75$, (e) $\beta = 0.99$, and (f) $\beta = 1.25$. For $\beta = 1.25$, the form of potential is a combination of expulsive harmonic oscillator and multi-color optical lattice given by Eq. (15). In Fig. 1a–f, each plot has two panels: the upper panel shows the density plot, and the lower panel indicates the corresponding trap profile. It is evident from the figure that for $\beta =0$, the potential is free space (Fig. 1a) and on increasing the magnitude of $\beta$, it becomes bi-periodic optical lattice potential. Till $\beta =0.25$, it remained single periodic potential (Fig. 1b) and subsequently the second smaller depth (frustration depth or disorder) occurs and its depth increases with the increase in magnitude of $\beta$ (Fig. 1c–f). It is clear from the figure that the condensate atomic density has a droplet profile in free space (Fig. 1a) and is identical to the results reported in the literature^9,10,40^.

In the case of trap transition from free space $\rightarrow$ optical lattice $\rightarrow$ BOL with increasing magnitude of $\beta$ from $0 \rightarrow 1.25$, the droplet profile smoothly transits from being a flat top density in free space to a bright soliton for BOL trap (Fig. 1b–f). Further, the fragmentation and compression of droplet width along with localization of atomic condensate density are also observed. The condensate density variation with changing potential depth is identical to the fragmentation of droplets observed by Zhou *et. al.* in optical lattice^55^. Here, the observation of droplet-to-soliton transition and Anderson-like localization in atomic condensate density profile can be attributed to the impact of the increase in potential depth and disorder of the BOL trap (Fig. 1c–f). With an increase in the potential depth, the droplet profile is fragmented into droplet lattices which gradually become bright solitons. Further, the increase in the disorder (with increasing $\beta$) results in the localization of atomic density which is the characteristic feature of disordered optical lattices like BOL^43–45^. The increase in $\beta$ magnitude leads to the generation of frustrated depths in the BOL trap and the change in the frustration depths decreases the effective barrier height between two lattice depths which enhances the quantum tunneling of BEC atoms towards the center of the trap leading to localization of atomic condensate density^32,33^. For better insight of this droplet to soliton transition, we plot in Fig. 1h the variation of EMF and BMF w.r.t. $\beta$. It is apparent from the figure that increases in $\beta$ result in increasing

the periodic strength of EMF and BMF interactions. Due to this, droplet profile fragments into bright solitons and illustrates Anderson-like localization. Further, the localization of solitons in a disordered optical lattice like BOL is well-studied in ultracold atoms literature^43,44^ and recently QDs to soliton transition is reported in the presence of harmonic confinement for binary BEC mixture^13^.

#### Estimation of temperature

Next, we estimate the variation in temperature for the changing potential depth of the BOL trap (by varying $\beta$) in Fig. 1g. According to the thermodynamic definition of temperature: $T = (\partial S/\partial E)^{-1}$, we evaluate the slope of entropy against the energy of the system for estimating the temperature of the system. Here, we calculate the entropy (S) of the system as: $S = -K_{B} \int _{-\infty }^{+\infty } |\psi (z, t)|^{2} ln |\psi (z, t)|^{2} \partial z$, where $K_{B}$ is the Boltzmann’s constant, $|\psi (z, t)|^{2}$ is the density of the condensate^33^. Further, the kinetic energy can be expressed as $E_{K}= \int _{-\infty }^{+\infty } |\partial \psi (z, t)/\partial z|^{2} \partial z$. In Fig. 1g, we illustrate the variation of *T* with respect to the $\beta$ by numerically evaluating the differential changes of entropy against energy. It is apparent from the figure that till $\beta$ changes from $0 \rightarrow 0.25$, the temperature of the system remains greater than zero (positive temperature). But as $\beta$ changes from $0.25 \rightarrow 1.25$, the frustration depth starts to appear in BOL trap (Fig. 1b–f) then the temperature of the system becomes negative (negative temperature). Here, the frustration depth is acting as the upper bound of energy level and leads to the generation of $T<0$ state. In Fig. 1h, we illustrate the profile of EMF and BMF for the changing magnitude of $\beta$ with the same physical parameter values and their magnitude remains comparable to each other throughout the region. Thus, from the constructed analytical model, we reveal a non-trivial transition of positive to negative temperature in the droplet phase and droplet to soliton transition by changing the potential depth or disorder in BOL confinement. This is one of the main results of the article.

**Figure 2:**
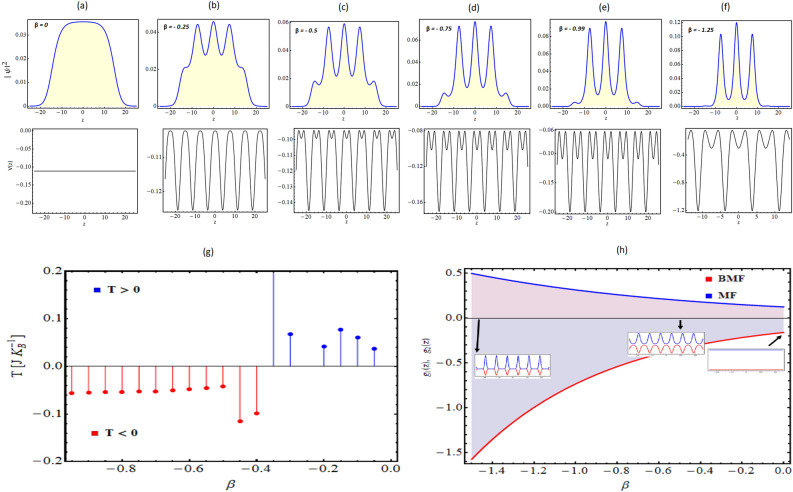
Condensate density patterns and corresponding BOL trap with $M = 0$ and $\beta \ne 0$: (**a**) $\beta = 0$ (free space); (**b**) $\beta = -0.25$, (**c**) $\beta = -0.5$, (**d**) $\beta = -0.75 0$, (**e**) $\beta = -0.99$, and (**f**) $\beta = -1.25$. Each plot of (**a**–**f**) has two panels: the upper panel shows the density plot, and the lower panel indicates the corresponding trap profile. (**g**) The figure depicts the estimate of temperature ($J K_{B}^{-1}$) with respect to the potential depth of BOL ($\beta$) and illustrates positive to negative temperature transition. (**h**) The profile of EMF and BMF is plotted by changing $\beta$ for $z=0$. Here, the magnitude of physical parameters: $\gamma _{0}=1$, $k=0.84$, $G_{1}=-1$, $G_{2}=0.999999$, $E=-2/9$. The spatial coordinate is scaled by the oscillator length. Figures are plotted using MATLAB R2020b (Master License 31349846) and Mathematica version 1.5.1.2021061827, Wolfram Research, Inc.

#### BOL symmetry impact

In the Fig. 2, we investigate the impact of BOL potential symmetry on the droplet-to-soliton transition and temperature of the considered system. For that purpose, we illustrate the condensate density patterns and corresponding BOL trap with $M = 0$ and $\beta < 0$: (a) $\beta = 0$ (free space); (b) $\beta = -0.25$, (c) $\beta = -0.5$, (d) $\beta = -0.75 0$, (e) $\beta = -0.99$, and (f) $\beta = -1.25$. Figure 2a–f has two panels: the upper panel shows the condensate density plot, and the lower panel indicates the corresponding external trap profile. In comparison to $\beta >0$ case in Fig. 1, the position of frustrated depth is shifted by half-wavelength in Fig. 2 BOL trap with $\beta <0$. This results in the positioning of droplet density maxima at $z=0$. Thus, by tuning the symmetry of the trap, one can control the positioning of droplet density maxima and minima locations. Further, similar to $\beta >0$ case, we observe the localization of free space droplet density to bright soliton by changing the magnitude of $\beta$ from $0 \rightarrow$ $-1.25$. In Fig. 2g, we study the variation of temperature ($J K_{B}^{-1}$) for the changing potential depth of BOL ($\beta$) and observe that decreasing $\beta$ results into positive to negative temperature transition similar to $\beta >0$ case. The decrease in the magnitude of $\beta$ from $0 \rightarrow -1.25$ leads to the formation of frustration depth in the chosen trap (Fig. 2) and this creates an upper bound in energy resulting in negative temperature. Figure 2h depicts EMF and BMF profiles with respect to the $\beta$ with the same physical parameter values.

#### Confirmation of droplet to soliton crossover

To confirm the observation of droplet-to-soliton transition, the energy of the system can be regarded as an important physical parameter. We calculate the total energy of the system:

21 $$\begin{aligned} \mu= & {} \int _{-\infty }^{+\infty } \left[ \frac{1}{2} \left( \frac{\partial \psi }{\partial z} \right) ^{2} + \psi ^{*} \nonumber \right. \\{} & {} \left. \left( \left[ -( \beta ^{2} k^{2})/16 \right] \cos (2 k \, \xi ) + \left[ (\beta k^{2}/4) + E \beta \right] \cos (k \, \xi ) \psi - g_1(z, t) |\psi | \psi + g_2(z, t)|\psi |^2 \psi \right) \right] . \end{aligned}$$

**Figure 3:**
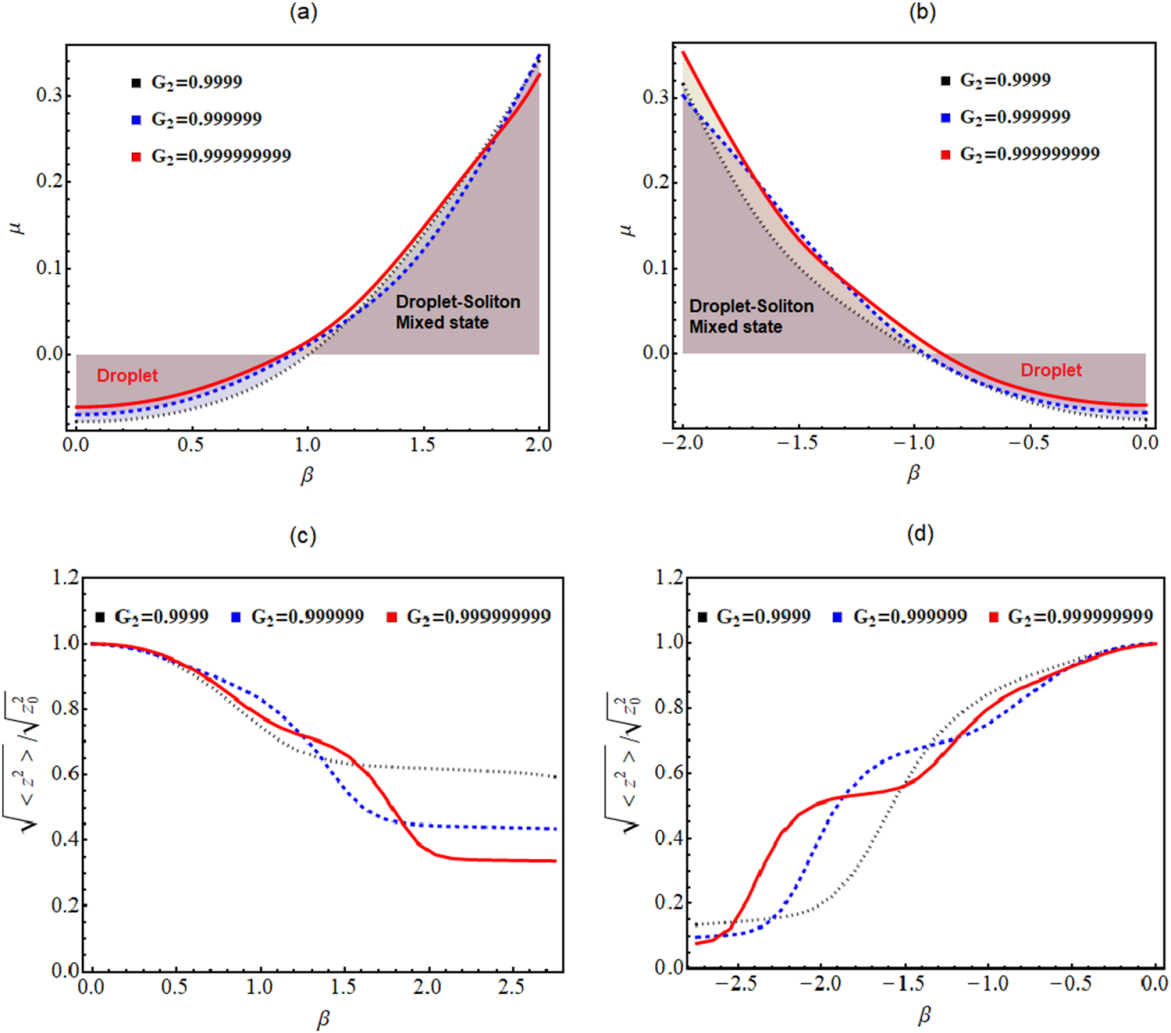
(**a**,**b**) Change in energy w.r.t to the variation of the BOL potential depth ($\beta$) are plotted here for different EMF interactions strengths. (**c**,**d**) Variation of the rms size of the droplets with the tuning of $\beta$. Here, solid red line, blue dashed, and black dotted lines denote the energy variation as a function of BOL potential strengths for $G_{2}=0.999999999$, 0.999999, and 0.9999, respectively. Further, the magnitude of physical parameters: $\gamma _{0}=1$, $k=0.84$, $G_{1}=-1$, $E=-2/9$. The spatial coordinate is scaled by the oscillator length.

Figure 3a illustrates the variation of total energy corresponding to the increase in the strength of BOL potential depth ($\beta$) for different magnitudes of EMF interaction strengths. Here, solid red line, blue dashed, and black dotted lines denote the energy variation as a function of BOL potential strengths for $G_{2}=0.999999999$, 0.999999, and 0.9999, respectively. In figure, the magnitude of physical parameters are: $\gamma _{0}=1$, $k=0.84$, $G_{1}=-1$, $E=-2/9$. The figure clearly illustrates that the energy is negative till $\beta < 0.9$ which indicates the accumulation of droplet-like bound pairs. In the vicinity of $0.9< \beta <1$, the energy crosses the zero line which indicates the breakdown of droplet-like bound pairs and results in the formation of solitons. For $\beta <0$, we plot the energy variation in Fig. 3b and observe the region of $-1< \beta <-0.9$ as the point for transition from droplet-to-soliton. We plotted (Fig. 3a,b) for ascertaining that this transition is a quantum phase transition (QPT) or crossover

for $\beta \rightarrow \pm 2$. As for the QPT, one observes a sudden change in the ground state of the many-body system for the case when a controlling parameter like $\beta$ of the Hamiltonian crosses a critical value. However, in Fig. 3a,b doesn’t illustrate any abrupt change in the energy ($\mu$) of the system. Thus, the transition appears to be gradual and termed as a droplet-to-soliton crossover.

Next, we depict the size variation in the droplet to soliton transition by estimating the root-mean-square (rms) size of the condensate $(\sqrt{<z^{2}>})$ with respect to $\beta$ in Fig. 3c,d. The rms size is plotted in the units of the rms size $(\sqrt{<z_{0}^{2}>})$ for $\beta =0$. The figure clearly indicates that the increase in EMF interaction strength ($0.9999 \rightarrow 0.999999999$) results in more decrease in the droplet size of the condensate as we illustrate the variation for $G_{2}=0.999999999$ (solid red line), 0.999999 (blue dashed) and 0.9999 (black dotted) lines, respectively. Here, we have considered the identical magnitude of physical parameters like above-mentioned cases: $\gamma _{0}=1$, $k=0.84$, $G_{1}=-1$, $E=-2/9$. Further, we observe that the smaller magnitude of $\beta$ allows the larger pair size with an asymptotic limit tending to 1 indicating the formation of droplets, and gradually the pair size smoothly decreases as the

**Figure 4:**
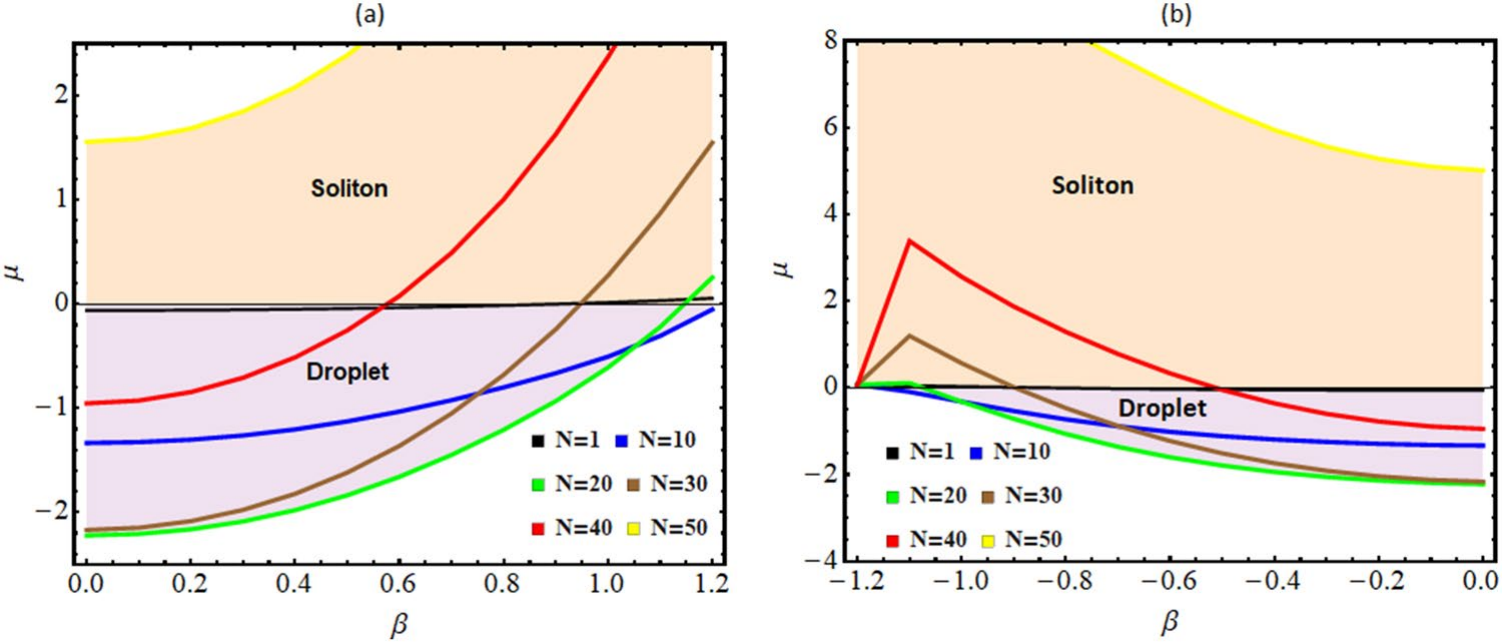
The figure illustrates the energy as a function of particle ($N=1$, 10, 20, 30, 40 and 50) and the BOL potential depth $\beta$ with (**a**) $0 < \beta \le 1.2$, and (**b**) $-1.2 \le \beta <0$. Here, the magnitude of physical parameters: $\gamma _{0}=1$, $k=0.84$, $G_{1}=-1$, $G_{1}=-0.999999999$, $E=-2/9$. A clear separation of droplet and soliton phase is indicated with increasing magnitude of *N* and $\beta$.

the potential strength of the BOL trap is increased. The rms size decrease in between the $\beta =0.8$ to 1.2 (Fig. 3c) or $\beta =-1.2$ to $-0.8$ (Fig. 3d) might be suggestive of an equilibrium of droplet-soliton mixture. Further increase in the $\beta$ leads to the total destruction of droplets as rms size becomes less than 1/5 th of the droplet size. The results of Fig. 3c,d complements the findings of Fig. 3a,b.

Finally, we construct a phase diagram in a space between the total energy and the particle number with respect to $\beta$ for (a) $0 < \beta \le 1.2$, and (b) $-1.2 \le \beta <0$ in the Fig. 4. For this, we calculate the energy for different particle number ($N=1$, 10, 20, 30, 40 and 50) with changing magnitude of $\beta$. In the figure, the light purple region corresponds to the negative energy region indicating the existence of the bound pairs and droplet formation. The light orange region describes the unbound solitonic region and corresponds to a region of droplet-to-soliton crossover where the droplets are breaking down into solitons. It is apparent from the figure that with an increase in the number of particles, the droplet-to-soliton transition occurs at smaller magnitudes of $\beta$ and the possibility of droplet phase formation reduces with an increase in the particle number. We can infer that in the region where the energy is negative for a relatively higher value of $\beta$, the energy of the system behaves like latent energy where a mixture of droplets and soliton coexist. In conclusion, depending upon the number of particles, the magnitude of $\beta$ varies at which the droplet-to-soliton transition occurs.

### Dynamics of quantum droplets in expulsive-BOL confinement

In this section, we investigate the generation of QDs and droplet-to-soliton transition in the time and space-modulated expulsive harmonic oscillator and BOL confinements. In order to construct these traps, we choose, $M \ne 0$, $\gamma _{0}=1$, and $\beta \ne 0$ in the potential Eq. (17) which results into the confinement form: $V(z,t) \simeq -\frac{1}{2} M^{2} z^{2}+ \gamma ^{2}_{0} Sech^{2}(M t) \left[ -\frac{\beta ^{2} k^{2} }{16} \right] \cos (2 k \, \xi ) + \gamma ^{2}_{0} Sech^{2}(M t) \left[ \frac{\beta k^{2}}{4} + E \beta \right] \cos (k \, \xi )$, with $\xi (z,t)= \gamma (t)z$. The corresponding BMF and EMF nonlinearities takes the form: $g_{1}(x,t)= (G_{1}/2 \sqrt{\gamma _{0} Sech(M t)}) exp[\beta \cos (k \gamma _{0} Sech(M t)z)]^{\frac{3}{2}}$, $g_{2}(x,t)=(G_{2} \gamma _{0} Sech(M t)/2 ) exp[\beta \cos (k \gamma _{0} Sech(M t)z)]^{3}$, respectively. Thus, the complete form of the wavefunction can be written from Eq. (18) as:

22 $$\begin{aligned} \psi (z,t)=\sqrt{\frac{\gamma _{0} Sech(M t)}{exp[\beta \cos (k\,\, \gamma _{0} Sech(M t) z)]}} \frac{\frac{3 E}{G_{1}} \times exp \left[ i \left( M Sech[M t]^2 Tanh[M t] \times z^{2} + \left( \frac{\beta ^{2} k^{2}}{16} \right) t\right) \right] }{1+\sqrt{1-\frac{E}{\mu _{0}} \frac{ G_{2}}{ G_{1}^{2}} } \cosh (\sqrt{\text {-E}} (\int _{0}^{ \gamma _{0} Sech(M t) z} exp[\beta \cos (k \gamma _{0} Sech(M t) z)] \partial z'))}, \end{aligned}$$

where $$\mu _{0}=-2/9$$, $$E<0$$, $$G_1<0$$ and $$G_2>0$$.

**Figure 5:**
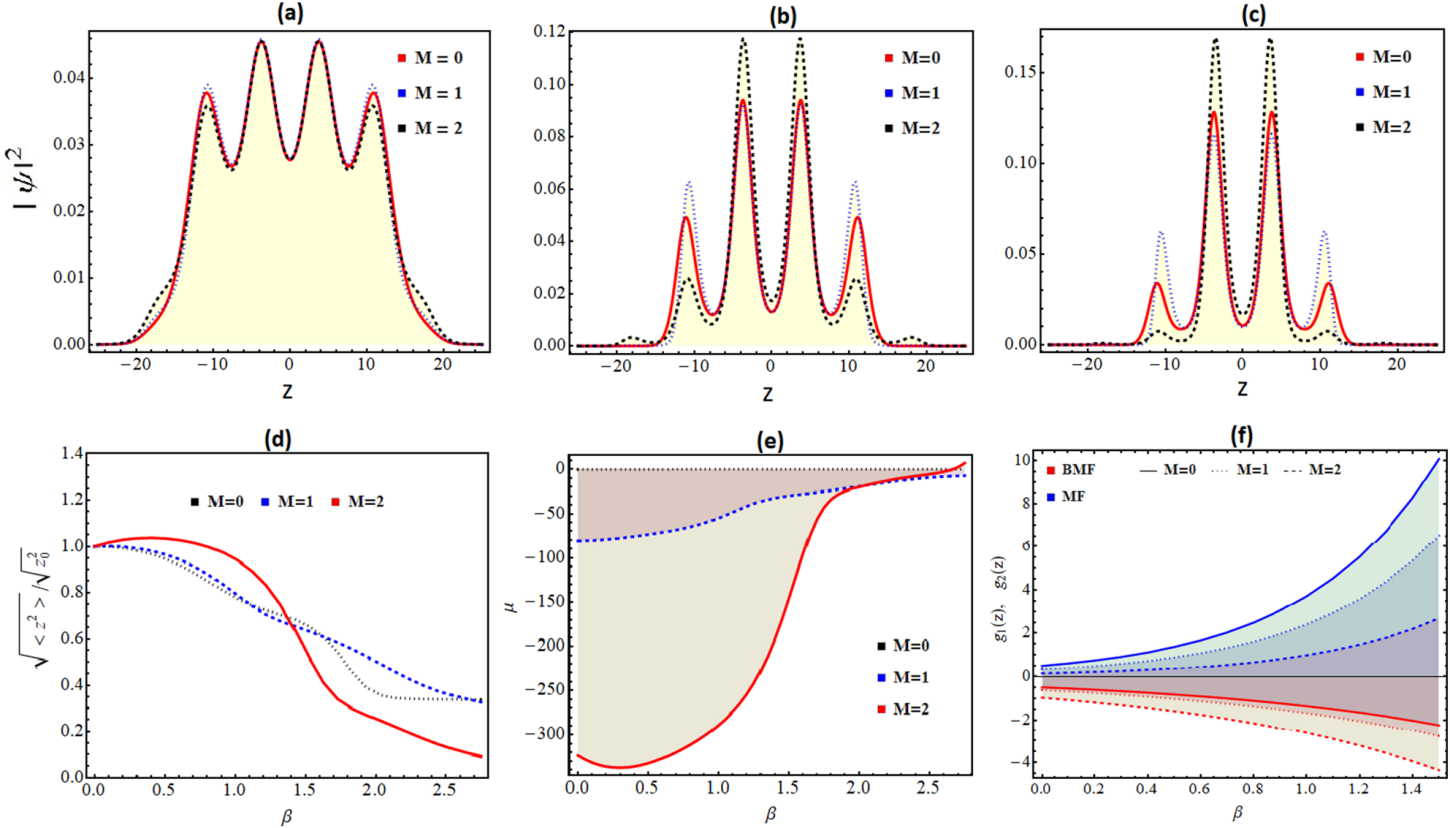
Variation of condensate density patterns for expulsive harmonic oscillator and BOL with respect to the *M* for (**a**) $\beta = 0.25$; (**b**) $\beta = 0.99$, (**c**) $\beta = 1.25$. (**d**) Variation of condensate rms size with $\beta$ and illustrating droplet to soliton crossover. (**e**) Change in energy with changing $\beta$ for $M=0$, 1,  and 2. (**f**) The profile of EMF and BMF is plotted with changing $\beta$. Here, the magnitude of physical parameters: $\gamma _{0}=1$, $k=0.84$, $G_{1}=-1$, $G_{2}=0.999999$, $E=-2/9$. The spatial coordinate is scaled by the oscillator length.

Using Eq. (22), we illustrate the variation of atomic density by changing the strength of the expulsive oscillator (*M*) for the combination of expulsive harmonic oscillator and BOL confinements in the Fig. 5a–c. Here, we have considered the magnitude of physical parameters: $\gamma _{0}=1$, $k=0.84$, $E=-2/9$, $G_{1}=-1$, $G_{2}=0.999999$, $t=1$ with (a) $\beta =0.25$, (b) $\beta =0.99$, and $\beta =1.25$, respectively. In this, $M=0$ (red line), $M=1$ (dashed blue line), and $M=2$ (dotted black line). It is evident from the figure that for $\beta =0.25$, a change in the strength of the expulsive trap has no significant impact on atomic condensate density for $M=0$, 1, or 2. This can be attributed to the fact for $\beta =0.25$, the atomic condensate density remains in the droplet phase (Fig. 5a). However, with $\beta$ changing from $0.25 \rightarrow 0.99 \rightarrow 1.25$ in Fig. 5b,c, there is significant difference in the localization of atomic density for three values of expulsive oscillator. Here, changing $\beta$ ensures that the system gradually moves from droplet to soliton phase, and an increase in expulsive oscillator strength results in faster localization of condensate atoms. This is due to the presence of *M* in potential depths of BOL and the increase of *M* results in the formation of frustration depth in the resultant confinement which leads to the observation of Anderson-like localization (Fig. 5a–c). For confirming the droplet-to-soliton crossover by tuning *M*, we plotted the variation of EMF and BMF profile w.r.t. $\beta$ for $M=0$ (solid line), 1 (dotted line) or 2 (dashed line) in Fig. 5f. It is apparent from the figure that increasing *M* results in an imbalance of EMF and BMF inter-atomic interactions leading to the solitonic phase.

For additional confirmation of the droplet-to-soliton crossover, we plot the rms size of the condensate $(\sqrt{<z^{2}>})$ in the units of the rms size $(\sqrt{<z_{0}^{2}>})$ with respect to $\beta$ for $M=0$, $\beta =0$ in Fig. 5d for the three values of expulsive-oscillator strength: $M=0$ (red solid line), 1 (blue dashed line) or 2 (black dotted line). It is evident from the figure that till the magnitude of $\beta =0.25$, the size of the droplet remains large, and with $\beta$ changing from $0.99 \rightarrow 1.25$, its size gradually reduces and the atomic condensate density reaches mixed droplet-soliton phase. For large $\beta$, the rms size of condensate becomes 1/5th of the largest values and represents the soliton phase. In Fig. 5d, the increase in the magnitude of *M* from $0 \rightarrow 2$ significantly controls the rate of reduction in the rms size of condensate and for large *M* values condensate fragments quickly into the smaller rms size and attains soliton phase. In order to corroborate this droplet to soliton transition, we also study the variation of energy: $\mu = \int _{-\infty }^{+\infty } \left[ \frac{1}{2} \left( \frac{\partial \psi }{\partial z} \right) ^{2} + \psi ^{*} \left( V(z,t) \psi - g_1(z, t) |\psi | \psi + g_2(z, t)|\psi |^2 \psi \right) \right]$ with changing $\beta$ for the chosen three expulsive oscillator strengths $M=0$, 1, and 2 in Fig. 5e. The figure clearly indicates that the presence of droplet phase i.e. $\mu < 0$ for the small magnitude of $\beta$ for all chosen values of *M* and

**Figure 6:**
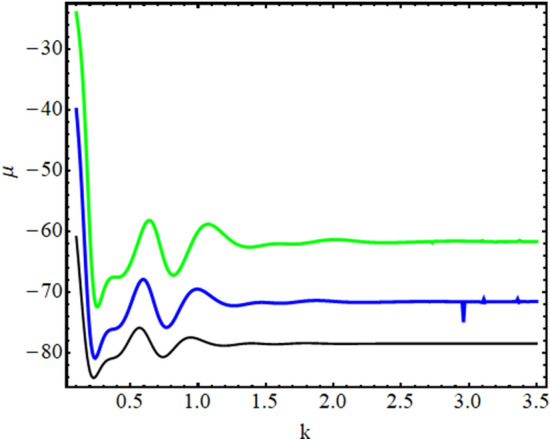
Variation of total energy ($\mu$) with respect to the lattice vector *k* for (**a**) $\beta = 0.25$ (black line); (**b**) $\beta = 0.5$ (blue line), (**c**) $\beta = 0.75$ (green line) in the presence of expulsive harmonic oscillator and BOL confinement. Here, the magnitude of physical parameters: $\gamma _{0}=1$, $M=1$, $k=0.84$, $G_{1}=-1$, $G_{2}=0.999999$, $E=-2/9$.

turns towards solitonic phase ($\mu >0$) for large $\beta$. In comparison to the previous case of BOL trap only, the amplitude of the expulsive oscillator (*M*) strengthens the droplet phase in the expulsive oscillator and BOL confinement and due to this condensate density remains in the droplet phase for a large magnitude of $\beta$.

Next in Table 1, we analyze the impact of expulsive oscillator strength on the temperature of the system and estimate the variation of temperature with changing magnitude of *M*. As discussed in the previous case, to calculate temperature $[T = (\partial S/\partial E)^{-1}]$, we evaluate the entropy (S): $S = -K_{B} \int _{-\infty }^{+\infty } |\psi (z, t)|^{2} ln |\psi (z, t)|^{2} \partial z$, and kinetic energy in reduced dimensionless model as: $E_{K}= \int _{-\infty }^{+\infty } |\partial \psi (z, t)/\partial z|^{2} \partial z$. Here, $K_{B}$ is Boltzmann’s constant, and $|\psi (z, t)|^{2}$ is the density of the condensate. It is apparent from the table that an increase in *M* leads to an increase in the negative temperature of the system in an expulsive-BOL trap. Further, in comparison to BOL trap (Figs. 1g and 2g), here temperature is negative with $\beta \ne 0$ whereas in BOL trap negative temperature is observed after $\beta$ reaches at critical values and frustration depth becomes significant in the BOL confinement. In this case, it is due to the creation of an upper bound in energy from the beginning due to the presence of an expulsive oscillator and BOL.

**Table 1:** Estimation of Temperature with changing *M* in expulsive-BOL confinement.

$\beta$	M = 1	M = 2
Temperature ($J K_{B}^{-1}$)		
0	0.00078837	0.000373552
0.1	− 0.012011	− 0.0419622
0.2	− 0.0277542	− 0.0275068
0.3	− 0.040899	− 0.0442314
0.4	− 0.053282	− 0.0671353
0.5	− 0.0645467	− 0.105269

Finally, we investigate the effect of an expulsive harmonic oscillator on the effective mass of localized mode (droplet and soliton) in the chosen confinements. It is already reported in the literature that the localized mode (soliton or droplet) formed under the action of the optical lattice potential may acquire an effective negative mass, in which case the expulsive potential actually traps the mode, rather than pushing it away^56^. Using the wavefunction solution (22), we estimate the motion of the droplet’s (or soliton’s) centre-of-mass coordinate [$Z_{P} = \int _{-\infty }^{+\infty } \psi ^{*} z \psi dz$] and momentum per atom [$p=i (\int _{-\infty }^{+\infty }( \partial \psi ^{*}/\partial z) \psi dz) /( \int _{-\infty }^{+\infty } \psi ^{*} \psi dz)$]^56^. For the constructed wavefunction solution, the magnitude of both quantities i.e. center of mass coordinate and momentum per atom is equal to zero. The reason for this result is connected with the choice of similarity variable ($\xi = \gamma (t) z$) in the constructed wavefunction solution. In this, $\gamma (t)$ is a positive definite function representing the inverse of the width of the localized solution, and the origin is the position of the center of mass^52^. If $\xi = \gamma (t) z + \delta (t)$, then for that case, the position of the center of mass will oscillate with $-\delta (t)/ \gamma (t)$^52^. Since the center of mass of the condensate is not moving, so the magnitude of velocity and momentum of localized modes (droplet or soliton) turns out to be zero. Next, to estimate the effective mass, we plot total energy $\mu$ with respect to lattice vector *k* in the Fig. 6. In the figure, the variation of total energy ($\mu$) with respect to the lattice vector *k* is illustrated for (a) $\beta = 0.25$ (black line); (b) $\beta = 0.5$ (blue line), (c) $\beta = 0.75$ (green line) for expulsive harmonic oscillator and BOL confinement. Here, the magnitude of physical parameters is identical to the previous cases: $\gamma _{0}=1$, $M=1$, $k=0.84$, $G_{1}=-1$, $G_{2}=0.999999$, $E=-2/9$. The figure indicates the negative effective mass [$\mu ''(k)$] of the localized mode in the expulsive harmonic oscillator and BOL in the region [0, 1.25] for each $\beta$.

## Summary

In summary, we construct an exact analytical model for 1D eGPE in the presence of an expulsive harmonic oscillator and BOL confinement which is suitable for the existence of quantum droplets at negative temperature. Interesting droplet to soliton crossover, Anderson-like localization of nonlinear excitations and the positive to negative temperatures transition observed in the binary BEC mixture in the presence of BOL and the combination of expulsive harmonic oscillator and BOL confinements. We begin by considering the 1D mass-balanced binary BEC mixture under the influence of competing repulsive cubic EMF and attractive quadratic BMF interactions. Utilizing the similarity transformation method, we calculate the non-trivial exact analytical form of the wavefunction, EMF, and BMF nonlinearities for an experimentally realizable form of trap configurations: (a) free space; (b) space-dependent BOL; and (c) space- and time-dependent expulsive harmonic oscillator and BOL confinements. The investigation of condensate dynamics reveals that the disorder and symmetry of the BOL trap control the transition from positive to negative temperature and the droplet-soliton crossover in chosen trap confinements. The affirmation of such crossover is performed by exploring the total energy and the rms size of the condensate bound pairs with changing potential depth and symmetry of the BOL trap. On that basis, we also construct a phase diagram in a space between the amplitude of BOL potential depth and particle number which reveals the formation of droplet and soliton phases. Further, for the combination of the expulsive harmonic oscillator and BOL trap, it is seen that the expulsive trap impact is insignificant on the atomic condensate density in the droplet phase and it becomes prominent in the soliton region. The magnitude of the expulsive oscillator strengthens the droplet phase and leads to an increase in the negative temperature of the system.

## Methods

We start by considering the 1D binary BEC mixture with equal atomic masses in presence of expulsive-BOL confinement under the BMF effects. In 1D geometry, the system is described by the following eGPE^10^:

23 $$\begin{aligned} i\frac{ \partial \psi }{\partial t} = -\frac{1}{2} \frac{ \partial ^2 \psi }{\partial z^2} - g_1(z, t) |\psi | \psi + g_2(z, t)|\psi |^2 \psi + V(z, t) \psi . \end{aligned}$$

For realizing negative temperature scenario, we choose a expulsive-BOL trap combination: $V(z,t) \simeq - \frac{1}{2}M(t)z^{2}+ V_{1}(t) \cos (2 k \, \xi ) + \left[ V_{2}(t) + E \beta \gamma ^{2}(t)\right] \cos (k \, \xi )$, where $\xi (z, t)= \gamma (t) z$, with $\gamma (t) \ne 0$, $-2/9 \le E < 0$, and $-1<\beta <1$. Utilizing the similarity transformation method, we begin by considering an ansatz solution and connecting the 1D eGPE with a solvable differential equation. This results in the consistency equations on the amplitude, phase, EMF, and BMF. By choosing the form of $\xi$, we solve these equations and obtain the general form of wavefunction solution.

## Data Availability

All data generated or analyzed during this study are included in this published article. It can be reproduced by utilizing the form of wavefunction and considered trap form.
